# Functional connectivity during tic suppression predicts reductions in vocal tics following behavior therapy in children with Tourette syndrome

**DOI:** 10.1017/S0033291723001940

**Published:** 2023-12

**Authors:** Simon Morand-Beaulieu, Michael J. Crowley, Heidi Grantz, James F. Leckman, Denis G. Sukhodolsky

**Affiliations:** 1 Department of Psychology, McGill University, Montreal, QC, Canada; 2 Child Study Center, Yale University School of Medicine, New Haven, CT, USA

**Keywords:** Behavior therapy, EEG, functional connectivity, tic suppression, Tourette syndrome

## Abstract

**Background:**

Comprehensive Behavioral Intervention for Tics (CBIT) is recommended as a first-line treatment for Tourette syndrome in children and adults. While there is strong evidence proving its efficacy, the mechanisms of reduction in tic severity during CBIT are still poorly understood. In a recent study, our group identified a functional brain network involved in tic suppression in children with TS. We reasoned that voluntary tic suppression and CBIT may share some mechanisms and thus we wanted to assess whether functional connectivity during tic suppression was associated with CBIT outcome.

**Methods:**

Thirty-two children with TS, aged 8 to 13 years old, participated in a randomized controlled trial of CBIT *v.* a treatment-as-usual control condition. EEG was recorded during tic suppression in all participants at baseline and endpoint. We used a source-reconstructed EEG connectivity pipeline to assess functional connectivity during tic suppression.

**Results:**

Functional connectivity during tic suppression did not change from baseline to endpoint. However, baseline tic suppression-related functional connectivity specifically predicted the decrease in vocal tic severity from baseline to endpoint in the CBIT group. Supplementary analyses revealed that the functional connectivity between the right superior frontal gyrus and the right angular gyrus was mainly driving this effect.

**Conclusions:**

This study revealed that functional connectivity during tic suppression at baseline predicted reduction in vocal tic severity. These results suggest probable overlap between the mechanisms of voluntary tic suppression and those of behavior therapy for tics.

## Introduction

Comprehensive Behavioral Intervention for Tics (CBIT) is recommended as a first-line treatment for Tourette syndrome (TS) in children and adults (Andrén et al., 2022; Pringsheim et al., 2019). The goal of CBIT is to increase the awareness of tics and premonitory urges and to engage in a behavior that is physically incompatible with the tic (Woods et al., 2008). While there is strong evidence proving the efficacy of CBIT (Piacentini et al., 2010; Wilhelm et al., 2012), the mechanisms underlying reductions in tic severity produced by this treatment are still poorly understood.

Recently, Essoe, Ramsey, Singer, Grados, and McGuire (2021a) reviewed the available evidence related to three potential mechanisms of behavior therapy for tics, namely reinforcement learning, habituation, and cognitive control. Reinforcement learning, which refers to the learning of an association between a behavior and an outcome, is thought to be linked with tic generation (Palminteri & Pessiglione, 2013). Previous CBIT research has hinted toward a possible role of different types of reinforcement learning, such as positive reinforcement (Essoe et al., 2021b) and reversal learning (McGuire et al., 2020). More research has been conducted on cognitive control, given that many cognitive control subprocesses are thought to play a role in CBIT: goal selection (awareness of internal and external contexts influencing tic expression), response selection (selection of competing responses), response inhibition (implementation of competing response inhibiting tic expression), and performance monitoring (making adjustments) (Essoe et al., 2021a; McGuire et al., 2022). While two studies reported a possible role of response inhibition and switching in CBIT (McGuire et al., 2022; Petruo et al., 2020), two large neuropsychological studies stemming from the initial randomized controlled trials (RCTs) of CBIT (Piacentini et al., 2010; Wilhelm et al., 2012) revealed that baseline cognitive control abilities did not predict CBIT outcome (Abramovitch et al., 2017; Chang et al., 2018). Our group also showed that EEG markers of response inhibition were unrelated to CBIT outcome (Morand-Beaulieu et al., 2022). Thus, specific aspects of cognitive control may play a role in CBIT but more research is definitely needed. Finally, there is some evidence supporting the role of habituation as a mechanism in some behavioral therapies such as exposure and response prevention (Hoogduin, Verdellen, & Cath, 1997; Verdellen et al., 2008), but it has not been investigated much in CBIT and so far its role remains unconclusive (Houghton et al., 2017).

Another process that may be linked with CBIT is voluntary tic suppression. Most people with TS can voluntarily suppress their tics, though tic suppressibility varies widely across individuals (Conelea et al., 2018; Ueda, Kim, Greene, & Black, 2021). While learning to control tics through CBIT is not the same as voluntarily suppressing them, there may be some parallels between both processes. Once CBIT is learned and put in application, it involves self-initiated techniques aimed toward controlling tic expression, similar to voluntary tic suppression. Both CBIT and tic suppression involve resisting a tic in response to a premonitory urge. Awareness of those urges is a core feature of CBIT (Woods et al., 2008) and is also associated with better tic suppression capacities (Matsuda et al., 2020). It has also been suggested that behavioral therapies for tics may bolster tic suppression capacities (Specht et al., 2014).

While the exact mechanism underlying tic suppression is still unclear, it could, just like behavior therapy, rely on a mix of reinforcement learning, cognitive control, and habituation. Prior research has found that tic suppression can be improved with positive reinforcement (Conelea et al., 2018). Additionally, some brain imaging studies (although conflicting findings exist; see van der Salm et al., 2018) suggested some overlap between the brain regions involved in tic suppression and those involved in response inhibition (Ganos et al., 2014; Peterson et al., 1998). Research on exposure and response prevention suggests that tic suppression may also partly operate through habituation, given that the person suppressing the tics must get used to and tolerate the urges to tic (Verdellen et al., 2008; Verdellen, Hoogduin, & Keijsers, 2007).

In a recent EEG study, our group identified a brain network in which functional connectivity was increased during tic suppression in children with TS (Morand-Beaulieu et al., 2023). Given that voluntary tic suppression and CBIT may share some common underpinnings, we wanted to assess whether functional connectivity in this network was associated with CBIT outcome. In treatments across several neurodevelopmental and mental health conditions, functional connectivity in networks that are associated with specific mechanisms relevant for that condition or treatment was found to either predict treatment outcome (Baumel et al., 2022; Cyr et al., 2020; Russman Block et al., 2022) or change after treatment (Baumel et al., 2022; Izadi-Najafabadi, Rinat, & Zwicker, 2022; Venkataraman et al., 2016).

Therefore, the first objective of this study was to test whether functional connectivity associated with tic suppression would change from baseline to endpoint. We predicted that functional connectivity in the tic suppression network would increase from baseline to endpoint in the CBIT group but not in the treatment-as-usual (TAU) condition. Our second objective was to test whether functional connectivity predicted reduction in tic severity following CBIT. We hypothesized that increased brain connectivity during tic suppression at baseline would predict greater decreases in tic severity at endpoint in the CBIT group.

## Methods

### Participants

Thirty-two children with TS, aged 8 to 13 years old, participated in a RCT of CBIT *v.* a TAU control condition (Morand-Beaulieu et al., 2022). Inclusion criteria consisted of (1) ages 8–14 years old; (2) DSM-IV-TR diagnosis criteria for TS or chronic tic disorder; (3) unmedicated or on stable medication for at least one month before initiating the study and throughout the duration of the study; (4) YGTSS Total Score > 14 or Total Score > 10 if only motor tics were present; and (5) fluent English speaker. Exclusion criteria consisted of (1) IQ < 80; (2) diagnosis of severe psychiatric disorder that could interfere with participation in the behavior therapy for tics (e.g. bipolar disorder or psychotic disorder); (3) presence of any psychiatric or psychosocial condition (e.g. depression or family discord) requiring initiation of treatment other than that provided in the current study (i.e. medication, family therapy) or change in current medication type or dose; (4) previous treatment with four or more sessions of habit reversal training/CBIT. The study was approved by Yale institutional review board and conducted according to the Declaration of Helsinki. Consent and assent were respectively obtained from parents and children prior to participation in the study. Families also received monetary compensation for their participation in the study assessment and therapy visits. Participants’ characteristics are presented in Table 1.

**Table 1.** Demographics and clinical characteristics by treatment group at baseline

	CBIT (*n* = 16)	TAU (*n* = 16)
Age in years, mean (s.d.)	11.4 (1.8)	11.3 (1.5)
Sex, number of boys (%)	14 (87.5)	13 (81.3)
Race, number (%)		
White	14 (87.5)	14 (87.5)
Black	1 (6.3)	1 (6.3)
Asian	1 (6.3)	1 (6.3)
Ethnicity, number of Hispanics (%)	2 (12.5)	0 (0)
Tic disorder diagnosis, number (%)		
Tourette syndrome	15 (93.8)	15 (93.8)
Persistent motor tic disorder	1 (6.3)	1 (6.3)
Other diagnoses, number (%)		
ADHD	9 (56.3)	8 (50.0)
OCD	3 (18.8)	2 (12.5)
ODD	3 (18.8)	3 (18.8)
Any anxiety disorder	3 (18.8)	4 (25.0)
Concomitant treatment status, number (%)		
Receiving psychotherapy	2 (12.5)	5 (31.3)
Receiving psychotropic medication	11 (68.8)	7 (43.8)

*Note:* ADHD, attention-deficit/hyperactivity disorder; CBIT, Comprehensive Behavioral Intervention for Tics; OCD, obsessive-compulsive disorder; ODD, oppositional defiant disorder; s.d., standard deviation.

### Procedures

#### Study design, randomization, and treatment

Full details pertaining to the structure of the RCT can be found in Morand-Beaulieu et al. (2022). In short, eligible participants were randomly assigned to receive 8 sessions of CBIT over a 10-week period or continue their current treatment plan as is (TAU group). In both treatment conditions, children maintained their ongoing treatment and appointments with their treating clinicians. Interventions were administered separately from the present study, based on the specific requirements of children and their parents, as well as the professional judgment of their treating clinician. Participants in both groups were allowed to receive their customary treatment and services, including, but not limited to, school-based services and individual child psychotherapy. Parents were requested to refrain from making any changes to their child's ongoing treatment or starting new treatments for the duration of the study.

#### EEG recordings

Continuous EEG was recorded at a 250 Hz sampling rate from 128 electrodes (HydroCel Geodesic Sensor Net) referenced online to the vertex electrode (Cz). We used a Net Amps 200 amplifier and Net Station Acquisition software version 4.2.1 (EGI, Inc.) to monitor signal acquisition. The sensor net was soaked in a potassium chloride solution prior to the recording session. Electrode impedance was assessed at or under 40 kΩ before recording. Data were online filtered with a 0.01 Hz high-pass filter and a 100 Hz low-pass filter.

#### Experimental task

EEG was recorded during three 2-min tic suppression sessions during which children were asked to suppress all tics. They were also asked to keep their eyes open while looking at the computer screen. Recordings took place in a dimly-lit room.

#### Outcome assessment

Changes in motor and vocal tic severity from baseline to endpoint were assessed by a blinded rater using the Yale Global Tic Severity Scale (Leckman et al., 1989). The YGTSS was administered by a trained clinician. It assesses the severity of tics over the past week. Motor and vocal tics are rated on 6-point scale (from 0 to 5) according to 5 dimensions: number, frequency, intensity, complexity, and interference. Thus, the motor and vocal subscale each range from 0 to 25 and can be combined to obtain the Total Tic Score.

### EEG signal treatment

#### EEG signal preprocessing

EEG recordings were preprocessed using the Maryland Analysis of Developmental EEG (MADE) pipeline (Debnath et al., 2020) running on Matlab 2020a. The MADE pipeline, which is based on EEGLAB's (Delorme & Makeig, 2004) functions and data structure, involves several steps which are fully described in the Supplement. Overall, signals were bandpass filtered from 1–50 Hz, bad channels were removed and interpolated, independent component analysis was performed to identify and remove non-neural artifact, continuous EEG recordings were epoched in 2-sec segments, residual artifacts in epoched EEG data were removed using a threshold rejection method, and data were re-referenced to the average of all electrodes. The number of valid 2-second epochs (CBIT: baseline: 131.0 ± 36.9, endpoint: 134.3 ± 44.8; TAU: baseline: 132.6 ± 37.6, endpoint: 122.2 ± 58.3) did not significantly differ across conditions or assessments [all *F*'s < 0.42, all *p* values > 0.40].

#### Source-based connectivity pipeline

Brain sources were reconstructed with minimum source imaging (wMNE) in Brainstorm (Tadel, Baillet, Mosher, Pantazis, & Leahy, 2011). Electrode positions were co-registered to the MNI-ICBM152 template using three reference points (nasion, left, and right preauricular points). The symmetric boundary element method implemented in OpenMEEG (Gramfort, Papadopoulo, Olivi, & Clerc, 2010) was used to compute a three-layered (scalp, outer skull, inner skull) head model. The diagonal of the noise covariance matrix was used for source reconstruction. Sources were projected onto the Desikan-Killiany atlas which consists of 34 regions of interest (ROI) per hemisphere.

The phase-locking value (PLV) was computed in Brainstorm and served as our measure of functional connectivity. The PLV reflects the absolute value of the mean phase difference between two signals (Aydore, Pantazis, & Leahy, 2013; Lachaux, Rodriguez, Martinerie, & Varela, 1999). It ranges from 0 to 1; larger values reflect stronger coupling between two signals. Prior research has shown that source-reconstructed connectivity using wMNE and PLV performed well against other methods (Hassan et al., 2017; Hassan, Dufor, Merlet, Berrou, & Wendling, 2014). The PLV was computed in the alpha band (8–13 Hz) with a Hilbert transform. The PLV was computed at the epoch level and was then averaged across all epochs. It was computed between all 68 ROI of the Desikan-Kiliany atlas yielding a 68 × 68 matrix for each participant at both baseline and endpoint. The subnetwork involved in tic suppression which we identified in a previous study (Morand-Beaulieu et al., 2023) was applied as a mask over connectivity matrices computed at baseline and endpoint. That subnetwork involved 29 connections between 28 ROIs (connections involved in this subnetwork are listed in online Supplementary Table S1). Baseline and endpoint mean functional connectivity across all connections involved in that subnetwork were used as the dependent variables in our analyses. Network visualization was performed with BrainNet Viewer (Xia, Wang, & He, 2013).

### Statistical analyses

To assess whether functional connectivity during tic suppression changed from baseline to endpoint, we conducted a repeated-measures ANOVA with Functional connectivity as the dependent variable, and with the between-subjects factor Treatment (CBIT and TAU) and the within-subjects factor Time (baseline and endpoint). We also conducted *t* tests (Bonferroni-corrected) on the individual connections comprised in the tic suppression subnetwork to assess if there were treatment effects that were specific to a given connection.

To test whether functional connectivity differently predicted reductions in motor and vocal tics, we included the YGTSS motor and vocal tic subscales as a factor in our prediction analyses. Thus, we conducted an ANCOVA on reductions in tic severity from baseline to endpoint with the between-subjects factor Treatment (CBIT and TAU) and the continuous predictor Baseline mean functional connectivity.

## Results

Results from the RCT have been published elsewhere (Morand-Beaulieu et al., 2022). Overall, CBIT had a positive impact on tic symptoms, with YGTSS total tic score decreasing from 23.8 ± 6.0 at baseline to 16.9 ± 4.9 at endpoint in the CBIT group. In the TAU group, average YGTSS total tic score went from 24.4 ± 5.0 at baseline to 24.9 ± 5.0 at endpoint (Table 2).

**Table 2.** Change in tic severity from baseline to endpoint

	CBIT (*n* = 16)		TAU (*n* = 16)		Time × Treatment interaction
	Baseline	Endpoint	Baseline	Endpoint	
YGTSS Total tic severity	23.8	16.9	24.4	24.9	*F*_(1,30)_ = 41.08, *p* < 0.001
YGTSS Motor tic severity	13.3	10.9	13.9	13.5	*F*_(1,30)_ = 7.71, *p* = 0.009
YGTSS Vocal tic severity	10.6	6.0	10.5	11.4	*F*_(1,30)_ = 27.20, *p* < 0.001

**Figure 1.**
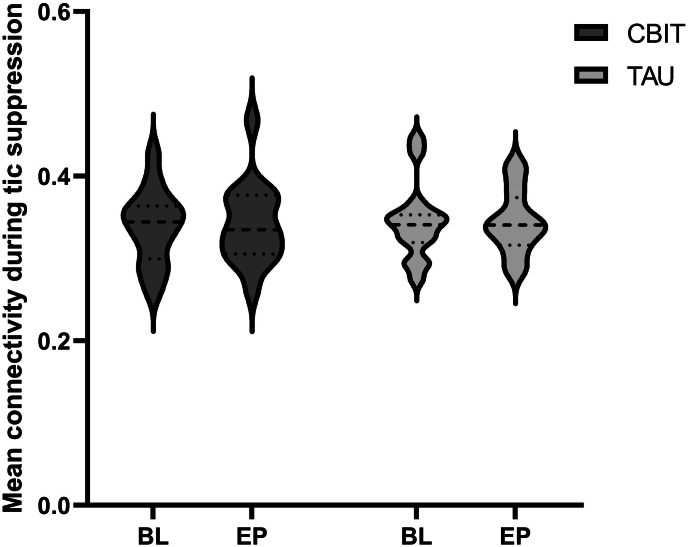
CBIT did not impact tic suppression-related functional connectivity. **BL**, baseline; CBIT, Comprehensive Behavioral Intervention for Tics; EP, endpoint; TAU, treatment-as-usual.

The first objective of this study was to assess if functional connectivity increased from baseline to endpoint in the CBIT group relative to TAU. Our analyses revealed no main effect of Time [*F*_(1,29)_ = 0.05, *p* = 0.82] and no Treatment by Time interaction [*F*_(1,29)_ = 0.06, *p* = 0.81], suggesting that there were no changes in mean functional connectivity during tic suppression from baseline to endpoint (Fig. 1). There were also no treatment-induced changes in functional connectivity for any connection comprised in the tic suppression network [all *t* values < 1.82, all *p* values > 0.078].

**Figure 2.**
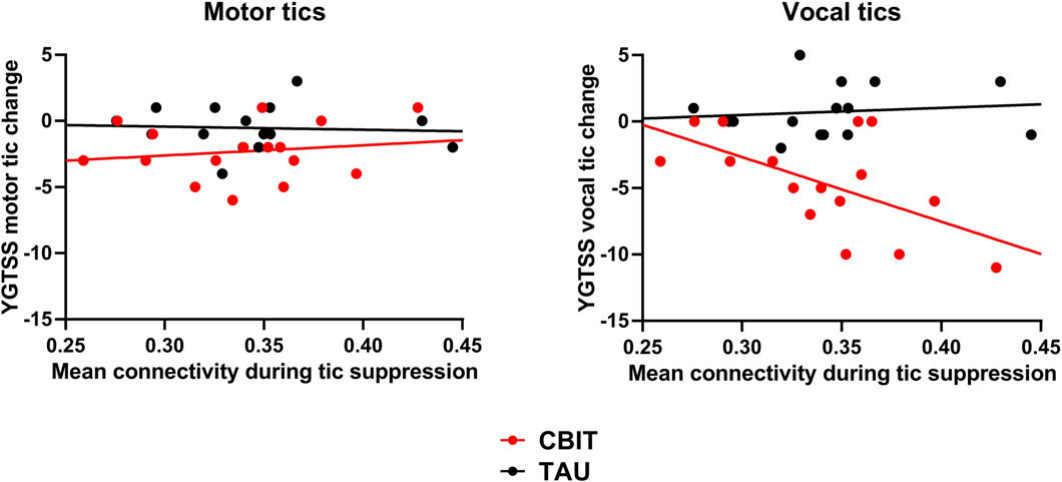
Prediction of CBIT outcome using baseline functional connectivity during tic suppression. Reduction in vocal tic severity following CBIT was predicted by mean connectivity during tic suppression at baseline. However, functional connectivity did not predict the decrease in motor tic severity. CBIT, Comprehensive Behavioral Intervention for Tics; TAU, treatment-as-usual; YGTSS, Yale Global Tic Severity Scale.

Regarding our second objective about predicting CBIT outcome using mean connectivity within the tic suppression network, the interaction between Treatment and Baseline mean functional connectivity was not significant [*F*_(1,27)_ = 3.14, *p* = 0.088]. Given that some studies have reported a differential impact on CBIT on motor *v.* vocal tics (Chen, Wang, Chang, & Hsueh, 2020; Yates et al., 2016), we decided to assess whether tic-related functional connectivity may differentially predict the impact of CBIT on motor and vocal tics. Thus, we conducted an ANCOVA on reductions in tic severity from baseline to endpoint with the between-subjects factor Treatment (CBIT *v.* TAU), the within-subjects factor Type of tics (motor *v.* vocal), and the continuous predictor Functional connectivity. We found a Treatment by Type of tics by Functional connectivity interaction [*F*_(1,27)_ = 4.89, *p* = 0.036]. Decomposition of this interaction revealed a Type of tics by Functional connectivity interaction in the CBIT group [*F*_(1,14)_ = 5.82, *p* = 0.030] but not in the TAU group [*F*_(1,13)_ = 0.12, *p* = 0.65]. In the CBIT group, functional brain connectivity during tic suppression at baseline predicted the decrease in vocal tic severity at endpoint [*R*^2^ = 0.35, *β* = −0.59, *t*(14) = −2.57, *p* = 0.015]. However, it did not predict the decrease in motor tic severity [*R*^2^ = 0.03, *β* = −0.16, *t*(14) = 0.62, *p* = 0.55] (Fig. 2).

**Figure 3.**
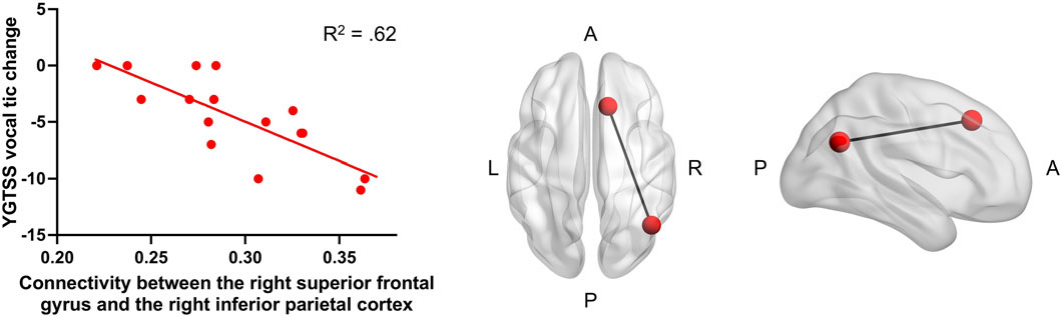
Association between decrease in vocal tic severity post-CBIT and superior frontal gyrus – inferior parietal cortex connectivity. Supplementary analyses were performed between single connections involved in the tic suppression subnetwork and vocal tic severity decreases in the CBIT group. One connection was significant according to the Bonferroni-corrected significance threshold. Within the CBIT group, decreases in vocal tic severity from baseline to endpoint were predicted by the functional connectivity between the right superior frontal gyrus and the right inferior parietal cortex. A, anterior; L, left; P, posterior; R, right.

Supplementary analyses were performed to identify whether specific connections within the tic suppression subnetwork were mainly responsible for the relationship between baseline functional connectivity during tic suppression and decrease in vocal tic severity post-CBIT. To identify potential associations between improvement in total and motor tic severity, which could have been masked by looking at the mean connectivity of the tic suppression subnetwork, supplementary analyses were conducted with the motor and total tic scales as well. Within the CBIT group, correlations were performed between each of the 29 connections involved in the tic suppression subnetwork and the decrease in vocal, motor, and total tic severity. A Bonferroni-corrected *p* value of *α* = 0.05/(29 × 3) = 0.00057 was used to account for the number of correlations. Only one correlation exceeded this threshold. There was a significant correlation between decrease in vocal tic severity and functional connectivity between the right superior frontal gyrus and the right inferior parietal cortex [*r*(14) = −0.79, *p* = 0.0003] (Fig. 3).

## Discussion

In the current study, we wished to assess how functional connectivity associated with tic suppression was related to CBIT response. EEG was recorded during three 2-min tic suppression sessions at baseline and endpoint. Brain sources were then reconstructed. We assessed functional connectivity within a subnetwork involved in tic suppression (Morand-Beaulieu et al., 2023) while children with TS were suppressing their tics.

Our analyses revealed that functional connectivity during tic suppression did not change from baseline to endpoint. In CBIT, tics are not directly suppressed but are replaced by competing responses (Piacentini et al., 2010; Woods et al., 2008). Thus, there is no exercise aimed toward enhancing voluntary tic suppression. While it has been proposed that CBIT may bolster tic suppression capacities (Specht et al., 2014), no study, to the best of our knowledge, has assessed whether voluntary tic suppression capacities were increased following CBIT. If CBIT does indeed have an impact on tic suppression capacities, this impact is not reflected on functional connectivity measures.

While functional connectivity during tic suppression is not altered by CBIT, it seems that it may predict its outcome. Indeed, baseline functional connectivity during tic suppression predicted reduction in vocal tic severity after CBIT. This suggests that strategies used in the voluntary suppression of vocal tics may be relevant during the course of the CBIT training. These results also suggest probable overlap between the mechanisms of voluntary tic suppression and those of behavior therapy for tics. However, in the present study, this effect was only present for vocal tics and not motor tics. In CBIT, competing responses for vocal tics generally consist of ‘controlled breathing’ (Woods et al., 2008). Such a technique may involve mechanisms that are more similar to those of voluntary tic suppression than those of competing responses for motor tics, which generally involves a movement or a response that is opposed or incompatible with the tic (Woods et al., 2008). Given the similarities between voluntary tic suppression and the management of vocal tics through CBIT, it is possible that children with increased functional connectivity during tic suppression were most likely to show larger decreases in vocal tic severity after CBIT. Another explanation may be related to the somatotopy of voluntary tic suppression. It is known that children are able to selectively inhibit particular tics, and in some contexts (e.g. in the classroom) they may inhibit vocal tics while continuing to have motor tics (Ganos et al., 2015). Therefore, if children in the current study were more used to suppress vocal than motor tics, the mechanism underlying this suppression may be potentiated by CBIT, which would in turn be associated with less severe vocal tics after treatment. Future studies should assess whether the same brain mechanisms are used to specifically suppress motor and vocal tics.

To the best of our knowledge, only one study directly assessed the link between tic suppression and CBIT. McGuire et al. (2022) reported that tic suppressibility (i.e. how much someone can suppress their tics) did not significantly predict the reduction in tic severity following CBIT, although the results were in the expected direction. Furthermore, they did not assess whether CBIT's effect on vocal tics differed from that of motor tics, and associations with vocal tic severity may have been masked by looking at total tic severity. While preliminary, our results suggest that changes in motor and vocal tic severity following CBIT may occur through different mechanisms. Few studies have separately assessed changes in motor and vocal tic severity during the course of therapy, but differences may exist (Shou et al., 2022). Our findings highlight the importance of providing separate measures of motor and vocal tic severity in clinical studies.

In our study, supplementary analyses revealed that the observed effect was mainly driven by the connection between the right superior frontal gyrus and the right inferior parietal cortex. In the Desikan-Killiany atlas, the anatomically-defined superior frontal gyrus encompasses the functionally-defined dorso-medial prefrontal cortex (dmPFC), and the inferior parietal cortex region includes both the inferior parietal gyrus and the angular gyrus (Desikan et al., 2006). The angular gyrus and the dmPFC are two important regions involved in the default mode network (Andrews-Hanna, Smallwood, & Spreng, 2014; Gusnard, Akbudak, Shulman, & Raichle, 2001). Prior research has suggested that the default mode network may constitute one of the neural substrate of premonitory urges (Ramkiran, Heidemeyer, Gaebler, Shah, & Neuner, 2019). Awareness of premonitory urges is important for both CBIT and tic suppression. Also, increased default mode network has been associated with reduced tic severity (Fan et al., 2018), which is consistent with our finding of larger decreases in vocal tic severity in those with increased baseline functional connectivity.

Finding predictors of treatment response is important from at least two perspectives: identifying individuals for whom CBIT may work well and understanding by which processes reductions in tic severity occur during CBIT. So far, few baseline characteristics may predict who is more likely to strongly benefit from CBIT. Sukhodolsky et al. (2017) found that greater baseline tic severity and treatment expectancy were associated with greater reductions in tic severity, whereas the presence of anxiety disorders and greater severity of premonitory urges predicted lesser reductions in tic severity following treatment. However, these effects were also seen in psychoeducation and supportive therapy and were not specific to CBIT. In a study where CBIT was combined with exposure and response prevention, larger obsessive-compulsive symptoms predicted larger decreases in tic severity after treatment (Nissen, Parner, & Thomsen, 2019). Likewise, not much is known about the mechanisms predicting the outcome of CBIT. Better performance in a neurocognitive measure combining interference control and cognitive flexibility has been shown greater reductions in tic severity (McGuire et al., 2022), suggesting that cognitive control may play a role in CBIT. Our study adds to this knowledge by identifying another process linked with treatment outcome. Such results are relevant for future studies aiming at better understanding the mechanisms of CBIT.

The findings of this study must be interpreted in the context of some limitations. First, our sample size was small, and our results need to be replicated in larger samples. Second, while using EEG allows the assessment of the synchronization of brain oscillations in a fast frequency band (alpha; 8–13 Hz), using fMRI would allow a more precise localization of the brain regions involved in tic suppression and CBIT response, compared with source-reconstructed EEG. Third, our hypotheses were based on potential parallels between tic suppression and behavior therapy. Thus, we decided to focus on a single subnetwork which we know is involved in tic suppression. Future studies should assess other known brain networks to better understand the role of functional connectivity in CBIT. Fourth, this study did not include a tic frequency count to assess the degree of tic suppressibility. This is in contrast with studies using the Tic Suppression Task from Woods and Himle (2004), which includes a behavioral index of tic suppression and reinforcement for not ticcing. Future studies investigating the brain correlates of tic suppression should use an observational system with established reliability to assess the degree of tic suppressibility (Black, Koller, & Black, 2021; Sturm et al., 2021). Finally, functional connectivity in the current study was specifically assessed during tic suppression. It would be interesting to assess functional connectivity during tasks designed to measure other plausible mechanisms of CBIT.

## Supporting information

Morand-Beaulieu et al. supplementary material Morand-Beaulieu et al. supplementary material

## Supplementary material

The supplementary material for this article can be found at https://doi.org/10.1017/S0033291723001940

## References

[ref1] Abramovitch, A., Hallion, L. S., Reese, H. E., Woods, D. W., Peterson, A., Walkup, J. T., … Wilhelm, S. (2017). Neurocognitive predictors of treatment response to randomized treatment in adults with tic disorders. Progress in Neuro-Psychopharmacology and Biological Psychiatry, 74, 9–14. 10.1016/j.pnpbp.2016.11.002. 27864156 PMC5330153

[ref2] Andrén, P., Jakubovski, E., Murphy, T. L., Woitecki, K., Tarnok, Z., Zimmerman-Brenner, S., … Verdellen, C. (2022). European clinical guidelines for Tourette syndrome and other tic disorders-version 2.0. Part II: Psychological interventions. European Child & Adolescent Psychiatry, 31(3), 403–423. 10.1007/s00787-021-01845-z. 34313861 PMC8314030

[ref3] Andrews-Hanna, J. R., Smallwood, J., & Spreng, R. N. (2014). The default network and self-generated thought: Component processes, dynamic control, and clinical relevance. Annals of the New York Academy of Sciences, 1316(1), 29–52. 10.1111/nyas.12360. 24502540 PMC4039623

[ref4] Aydore, S., Pantazis, D., & Leahy, R. M. (2013). A note on the phase locking value and its properties. Neuroimage, 74, 231–244. 10.1016/j.neuroimage.2013.02.008. 23435210 PMC3674231

[ref5] Baumel, W. T., Lu, L., Huang, X., Drysdale, A. T., Sweeny, J. A., Gong, Q., … Strawn, J. R. (2022). Neurocircuitry of treatment in anxiety disorders. Biomarkers in Neuropsychiatry, 6, 100052. 10.1016/j.bionps.2022.100052. PMC9222661 35756886

[ref6] Black, J., Koller, J., & Black, K. (2021). TicTimer web: Software for measuring tic suppression remotely [version 2; peer review: 2 approved]. F1000Research, 9, 1264. 10.12688/f1000research.26347.2. PMC7993402 33824720

[ref7] Chang, S. W., McGuire, J. F., Walkup, J. T., Woods, D. W., Scahill, L., Wilhelm, S., … Piacentini, J. (2018). Neurocognitive correlates of treatment response in children with Tourette's disorder. Psychiatry Research, 261, 464–472. 10.1016/j.psychres.2017.12.066. 29407718 PMC5809184

[ref8] Chen, C. W., Wang, H. S., Chang, H. J., & Hsueh, C. W. (2020). Effectiveness of a modified comprehensive behavioral intervention for tics for children and adolescents with Tourette's syndrome: A randomized controlled trial. Journal of Advanced Nursing, 76(3), 903–915. 10.1111/jan.14279. 31782167

[ref9] Conelea, C. A., Wellen, B., Woods, D. W., Greene, D. J., Black, K. J., Specht, M., … Capriotti, M. (2018). Patterns and predictors of tic suppressibility in youth with tic disorders. Frontiers in Psychiatry, 9, 188–188. 10.3389/fpsyt.2018.00188. 29875706 PMC5974106

[ref10] Cyr, M., Pagliaccio, D., Yanes-Lukin, P., Fontaine, M., Rynn, M. A., & Marsh, R. (2020). Altered network connectivity predicts response to cognitive-behavioral therapy in pediatric obsessive-compulsive disorder. Neuropsychopharmacology, 45(7), 1232–1240. 10.1038/s41386-020-0613-3. 31952071 PMC7235012

[ref11] Debnath, R., Buzzell, G. A., Morales, S., Bowers, M. E., Leach, S. C., & Fox, N. A. (2020). The Maryland analysis of developmental EEG (MADE) pipeline. Psychophysiology, 57(6), e13580. 10.1111/psyp.13580. 32293719 PMC12758016

[ref12] Delorme, A., & Makeig, S. (2004). EEGLAB: An open source toolbox for analysis of single-trial EEG dynamics including independent component analysis. Journal of Neuroscience Methods, 134(1), 9–21. 10.1016/j.jneumeth.2003.10.009. 15102499

[ref13] Desikan, R. S., Ségonne, F., Fischl, B., Quinn, B. T., Dickerson, B. C., Blacker, D., … Killiany, R. J. (2006). An automated labeling system for subdividing the human cerebral cortex on MRI scans into gyral based regions of interest. NeuroImage, 31(3), 968–980. 10.1016/j.neuroimage.2006.01.021. 16530430

[ref14] Essoe, J. K. Y., Ramsey, K. A., Singer, H. S., Grados, M., & McGuire, J. F. (2021a). Mechanisms underlying behavior therapy for Tourette's disorder. Current Developmental Disorders Reports, 8, 161–174. 10.1007/s40474-021-00225-1.

[ref15] Essoe, J. K. Y., Ricketts, E. J., Ramsey, K. A., Piacentini, J., Woods, D. W., Peterson, A. L., … McGuire, J. F. (2021b). Homework adherence predicts therapeutic improvement from behavior therapy in Tourette's disorder. Behaviour Research and Therapy, 140, 103844. 10.1016/j.brat.2021.103844. 33770556 PMC8026681

[ref16] Fan, S., van den Heuvel, O. A., Cath, D. C., de Wit, S. J., Vriend, C., Veltman, D. J., & van der Werf, Y. D. (2018). Altered functional connectivity in resting state networks in Tourette's disorder. Frontiers in Human Neuroscience, 12, 363–363. 10.3389/fnhum.2018.00363. 30279651 PMC6154258

[ref17] Ganos, C., Bongert, J., Asmuss, L., Martino, D., Haggard, P., & Münchau, A. (2015). The somatotopy of tic inhibition: Where and how much? Movement Disorders, 30(9), 1184–1189. 10.1002/mds.26188. 25786675

[ref18] Ganos, C., Kahl, U., Brandt, V., Schunke, O., Baumer, T., Thomalla, G., … Kuhn, S. (2014). The neural correlates of tic inhibition in Gilles de la Tourette syndrome. Neuropsychologia, 65, 297–301. 10.1016/j.neuropsychologia.2014.08.007. 25128587

[ref19] Gramfort, A., Papadopoulo, T., Olivi, E., & Clerc, M. (2010). OpenMEEG: Opensource software for quasistatic bioelectromagnetics. BioMedical Engineering OnLine, 9, 45. 10.1186/1475-925x-9-45. 20819204 PMC2949879

[ref20] Gusnard, D. A., Akbudak, E., Shulman, G. L., & Raichle, M. E. (2001). Medial prefrontal cortex and self-referential mental activity: Relation to a default mode of brain function. Proceedings of the National Academy of Sciences, 98(7), 4259. 10.1073/pnas.071043098. PMC31213 11259662

[ref21] Hassan, M., Dufor, O., Merlet, I., Berrou, C., & Wendling, F. (2014). EEG source connectivity analysis: From dense array recordings to brain networks. PLOS ONE, 9(8), e105041. 10.1371/journal.pone.0105041. 25115932 PMC4130623

[ref22] Hassan, M., Merlet, I., Mheich, A., Kabbara, A., Biraben, A., Nica, A., & Wendling, F. (2017). Identification of interictal epileptic networks from Dense-EEG. Brain Topography, 30(1), 60–76. 10.1007/s10548-016-0517-z. 27549639

[ref23] Hoogduin, K., Verdellen, C., & Cath, D. (1997). Exposure and response prevention in the treatment of Gilles de la Tourette's syndrome: Four case studies. Clinical Psychology & Psychotherapy, 4(2), 125–135. 10.1002/(SICI)1099-0879(199706)4:2<125::AID-CPP125>3.0.CO;2-Z.

[ref24] Houghton, D. C., Capriotti, M. R., Scahill, L. D., Wilhelm, S., Peterson, A. L., Walkup, J. T., … Woods, D. W. (2017). Investigating habituation to premonitory urges in behavior therapy for tic disorders. Behavior Therapy, 48(6), 834–846. 10.1016/j.beth.2017.08.004. 29029679 PMC5679290

[ref25] Izadi-Najafabadi, S., Rinat, S., & Zwicker, J. G. (2022). Brain functional connectivity in children with developmental coordination disorder following rehabilitation intervention. Pediatric Research, 91(6), 1459–1468. 10.1038/s41390-021-01517-3. 33934120 PMC9197764

[ref26] Lachaux, J.-P., Rodriguez, E., Martinerie, J., & Varela, F. J. (1999). Measuring phase synchrony in brain signals. Human Brain Mapping, 8(4), 194–208. 10.1002/(sici)1097-0193(1999)8:4<194::aid-hbm4>3.0.co;2-c. 10619414 PMC6873296

[ref27] Leckman, J. F., Riddle, M. A., Hardin, M. T., Ort, S. I., Swartz, K. L., Stevenson, J., & Cohen, D. J. (1989). The Yale global tic severity scale: Initial testing of a clinician-rated scale of tic severity. Journal of the American Academy of Child and Adolescent Psychiatry, 28(4), 566–573. 10.1097/00004583-198907000-00015. 2768151

[ref28] Matsuda, N., Nonaka, M., Kono, T., Fujio, M., Nobuyoshi, M., & Kano, Y. (2020). Premonitory awareness facilitates tic suppression: Subscales of the premonitory urge for tics scale and a new self-report questionnaire for tic-associated sensations. Frontiers in Psychiatry, 11, 592. 10.3389/fpsyt.2020.00592. 32719621 PMC7350852

[ref29] McGuire, J. F., Ginder, N., Ramsey, K., Essoe, J. K., Ricketts, E. J., McCracken, J. T., & Piacentini, J. (2020). Optimizing behavior therapy for youth with Tourette's disorder. Neuropsychopharmacology, 45(12), 2114–2119. 10.1038/s41386-020-0762-4. 32653895 PMC7547669

[ref30] McGuire, J. F., Sturm, A., Ricketts, E. J., Montalbano, G. E., Chang, S., Loo, S. K., … Piacentini, J. (2022). Cognitive control processes in behavior therapy for youth with Tourette's disorder. Journal of Child Psychology and Psychiatry, 63(3), 296–304. 10.1111/jcpp.13470. 34155637 PMC10696898

[ref31] Morand-Beaulieu, S., Crowley, M. J., Grantz, H., Leckman, J. F., Scahill, L., & Sukhodolsky, D. G. (2022). Evaluation of EEG biomarkers of comprehensive behavioral intervention for tics in children with Tourette syndrome. Clinical Neurophysiology. 10.1016/j.clinph.2022.07.500. 35987093

[ref32] Morand-Beaulieu, S., Wu, J., Mayes, L. C., Grantz, H., Leckman, J. F., Crowley, M. J., … Sukhodolsky, D. G. (2023). Increased alpha-band connectivity during tic suppression in children with Tourette syndrome revealed by source EEG analyses. Biological Psychiatry: Cognitive Neuroscience and Neuroimaging, 8(3), 241–250. 10.1016/j.bpsc.2021.05.001. 33991741 PMC8589865

[ref33] Nissen, J. B., Parner, E. T., & Thomsen, P. H. (2019). Predictors of therapeutic treatment outcome in adolescent chronic tic disorders. BJPsych Open, 5(5), e74. 10.1192/bjo.2019.56. 31409430 PMC6737514

[ref34] Palminteri, S., & Pessiglione, M. (2013). Reinforcement learning and Tourette syndrome. In D. Martino & A. E. Cavanna (Eds.), International review of neurobiology (Vol. 112, pp. 131–153). Cambridge, MA: Academic Press. 10.1016/B978-0-12-411546-0.00005-6. 24295620

[ref35] Peterson, B. S., Skudlarski, P., Anderson, A. W., Zhang, H., Gatenby, J. C., Lacadie, C. M., … Gore, J. C. (1998). A functional magnetic resonance imaging study of tic suppression in Tourette syndrome. Archives of General Psychiatry, 55(4), 326–333. 9554428 10.1001/archpsyc.55.4.326

[ref36] Petruo, V., Bodmer, B., Bluschke, A., Münchau, A., Roessner, V., & Beste, C. (2020). Comprehensive behavioral intervention for tics reduces perception-action binding during inhibitory control in Gilles de la Tourette syndrome. Scientific Reports, 10(1), 1174. 10.1038/s41598-020-58269-z. 31980733 PMC6981113

[ref37] Piacentini, J., Woods, D. W., Scahill, L., Wilhelm, S., Peterson, A. L., Chang, S., … Walkup, J. T. (2010). Behavior therapy for children with Tourette disorder: A randomized controlled trial. JAMA, 303(19), 1929–1937. 10.1001/jama.2010.607. 20483969 PMC2993317

[ref38] Pringsheim, T., Okun, M. S., Müller-Vahl, K., Martino, D., Jankovic, J., Cavanna, A. E., … Piacentini, J. (2019). Practice guideline recommendations summary: Treatment of tics in people with Tourette syndrome and chronic tic disorders. Neurology, 92(19), 896–906. 10.1212/wnl.0000000000007466. 31061208 PMC6537133

[ref39] Ramkiran, S., Heidemeyer, L., Gaebler, A., Shah, N. J., & Neuner, I. (2019). Alterations in basal ganglia-cerebello-thalamo-cortical connectivity and whole brain functional network topology in Tourette's syndrome. NeuroImage: Clinical, 24, 101998. 10.1016/j.nicl.2019.101998. 31518769 PMC6742843

[ref40] Russman Block, S., Norman, L. J., Zhang, X., Mannella, K. A., Yang, H., Angstadt, M., … Fitzgerald, K. D. (2022). Resting-state connectivity and response to psychotherapy treatment in adolescents and adults with OCD: A randomized clinical trial. American Journal of Psychiatry, 180(1), 89–99. 10.1176/appi.ajp.21111173. 36475374 PMC10956516

[ref41] Shou, S., Li, Y., Fan, G., Zhang, Q., Yan, Y., Lv, T., & Wang, J. (2022). The efficacy of cognitive behavioral therapy for tic disorder: A meta-analysis and a literature review. Frontiers in Psychology, 13, 851250. 10.3389/fpsyg.2022.851250. 35401364 PMC8987272

[ref42] Specht, M. W., Nicotra, C. M., Kelly, L. M., Woods, D. W., Ricketts, E. J., Perry-Parrish, C., … Walkup, J. T. (2014). A comparison of urge intensity and the probability of tic completion during tic freely and tic suppression conditions. Behavior Modification, 38(2), 297–318. 10.1177/0145445514537059. 24924158

[ref43] Sturm, A., Ricketts, E. J., McGuire, J. F., Lerner, J., Lee, S., Loo, S. K., … Piacentini, J. (2021). Inhibitory control in youth with Tourette's disorder, attention-deficit/hyperactivity disorder and their combination and predictors of objective tic suppressibility. Psychiatry Research, 304, 114163. 10.1016/j.psychres.2021.114163. 34411767 PMC8809367

[ref44] Sukhodolsky, D. G., Woods, D. W., Piacentini, J., Wilhelm, S., Peterson, A. L., Katsovich, L., … Scahill, L. (2017). Moderators and predictors of response to behavior therapy for tics in Tourette syndrome. Neurology, 88(11), 1029–1036. 10.1212/WNL.0000000000003710. 28202705 PMC5384839

[ref45] Tadel, F., Baillet, S., Mosher, J. C., Pantazis, D., & Leahy, R. M. (2011). Brainstorm: A user-friendly application for MEG/EEG analysis. Computational Intelligence and Neuroscience, 2011, 879716. 10.1155/2011/879716. 21584256 PMC3090754

[ref46] Ueda, K., Kim, S., Greene, D. J., & Black, K. J. (2021). Correlates and clinical implications of tic suppressibility. Current Developmental Disorders Reports, 8(2), 112–120. 10.1007/s40474-021-00230-4. 34178574 PMC8224814

[ref47] van der Salm, S. M. A., van der Meer, J. N., Cath, D. C., Groot, P. F. C., van der Werf, Y. D., Brouwers, E., … Tijssen, M. A. J. (2018). Distinctive tics suppression network in Gilles de la Tourette syndrome distinguished from suppression of natural urges using multimodal imaging. NeuroImage: Clinical, 20, 783–792. 10.1016/j.nicl.2018.09.014. 30268027 PMC6169325

[ref48] Venkataraman, A., Yang, D. Y., Dvornek, N., Staib, L. H., Duncan, J. S., Pelphrey, K. A., & Ventola, P. (2016). Pivotal response treatment prompts a functional rewiring of the brain among individuals with autism spectrum disorder. Neuroreport, 27(14), 1081–1085. 10.1097/wnr.0000000000000662. 27532879 PMC5007196

[ref49] Verdellen, C. W., Hoogduin, C. A., Kato, B. S., Keijsers, G. P., Cath, D. C., & Hoijtink, H. B. (2008). Habituation of premonitory sensations during exposure and response prevention treatment in Tourette's syndrome. Behavior Modification, 32(2), 215–227. 10.1177/0145445507309020. 18285507

[ref50] Verdellen, C. W., Hoogduin, C. A., & Keijsers, G. P. (2007). Tic suppression in the treatment of Tourette's syndrome with exposure therapy: The rebound phenomenon reconsidered. Movement Disorders: Official Journal of the Movement Disorder Society, 22(11), 1601–1606. 10.1002/mds.21577. 17534958

[ref51] Wilhelm, S., Peterson, A. L., Piacentini, J., Woods, D. W., Deckersbach, T., Sukhodolsky, D. G., … Scahill, L. (2012). Randomized trial of behavior therapy for adults with Tourette syndrome. Archives of General Psychiatry, 69(8), 795–803. 10.1001/archgenpsychiatry.2011.1528. 22868933 PMC3772729

[ref52] Woods, D. W., & Himle, M. B. (2004). Creating tic suppression: Comparing the effects of verbal instruction to differential reinforcement. Journal of Applied Behavior Analysis, 37(3), 417–420. 10.1901/jaba.2004.37-417. 15529900 PMC1284518

[ref53] Woods, D. W., Piacentini, J., Chang, S., Deckersbach, T., Ginsburg, G., Peterson, A., … Wilhelm, S. (2008). Managing Tourette syndrome: A behavioral intervention for children and adults - therapist guide. New York, NY: Oxford University Press.

[ref54] Xia, M., Wang, J., & He, Y. (2013). BrainNet viewer: A network visualization tool for human brain connectomics. PLOS ONE, 8(7), e68910. 10.1371/journal.pone.0068910. 23861951 PMC3701683

[ref55] Yates, R., Edwards, K., King, J., Luzon, O., Evangeli, M., Stark, D., … Murphy, T. (2016). Habit reversal training and educational group treatments for children with Tourette syndrome: A preliminary randomised controlled trial. Behaviour Research and Therapy, 80, 43–50. 10.1016/j.brat.2016.03.003. 27037483

