# Supplementary material for: Functional connectivity during tic suppression predicts reductions in vocal tics following behavior therapy in children with Tourette syndrome
Source: Psychol Med. 2023 Jul 24;53(16):7857–64. doi: 10.1017/S0033291723001940 (PMC10755221; doi:10.1017/S0033291723001940)
Supplement: Morand-Beaulieu et al. supplementary material [file S0033291723001940sup001.docx]

**Functional connectivity during tic suppression predicts reductions in vocal tics following behavior therapy in children with Tourette syndrome**

Simon Morand-Beaulieu, Michael J. Crowley, Heidi Grantz, James F. Leckman, Denis G. Sukhodolsky

***Supplement***

**Supplementary methods**

*EEG preprocessing*

The first step of the Maryland Analysis of Developmental EEG (MADE) (Debnath et al., 2020) pipeline consists in offline filtering of the data using EEGLAB’s firfilt plugin. Continuous data were filtered with a Hamming window finite impulse response (FIR) filter, with a 1 Hz high-pass filter (0.3 Hz transition width) and a 50 Hz low-pass filter (10 Hz transition width). The stopband attenuation was 53 dB. Then, the channel_properties function from EEGLAB’s FASTER plugin (Nolan, Whelan, & Reilly, 2010) was used to identify bad channels according to three-values: Hurst exponent, correlation with other channels, and channel variance. After removal of bad channels, we used an independent component analysis (ICA) method to remove non-neural artifacts such as blinks, saccades, and muscle artifacts, which can be especially prevalent in children with TS. Since ICA performs better on data filtered with higher high-pass filter, the ICA was performed on a copy of the recordings, which is high-pass filtered at 1 Hz and epoched into 1-second segments. At this stage, epochs with very low/high amplitude epochs (± 1000 μV) and excessive EMG activity (exceeding -30 and 100 dB between 20-40 Hz) were identified. Channels containing more than 20% of these epochs, suggesting bad channels missed by FASTER, were removed from the copied and the original datasets. These bad epochs were then removed from the copied dataset. Then, the ICA was performed on the copied dataset using EEGLAB’s runica function. ICA weights were transferred to the original dataset. Artifacted independent components were identified with a modified version of EEGLAB’s ADJUST plugin (Mognon, Jovicich, Bruzzone, & Buiatti, 2011), which is adapted for EEG recordings in children (Leach et al., 2020). Artifacted independent components were subtracted from the original dataset. Continuous data was then epoched into 2-seconds segments with 50% overlap. Following data cleaning with ICA, residual artifact were removed with a threshold rejection method. For frontal electrodes near the eyes (1, 8, 14, 21, 25, 32), any epoch with voltage exceeding ±100 μV was removed. For other electrodes, epochs with more than 10% of electrodes exceeding ±100 μV were removed. For remaining epochs, individual channels were interpolated with a spherical spline procedure at the epoch level if they exceeded ±100 μV. Channels that were removed using the FASTER plugin were then interpolated, also with a spherical spline procedure. Finally, electrodes were re-referenced to the average reference.


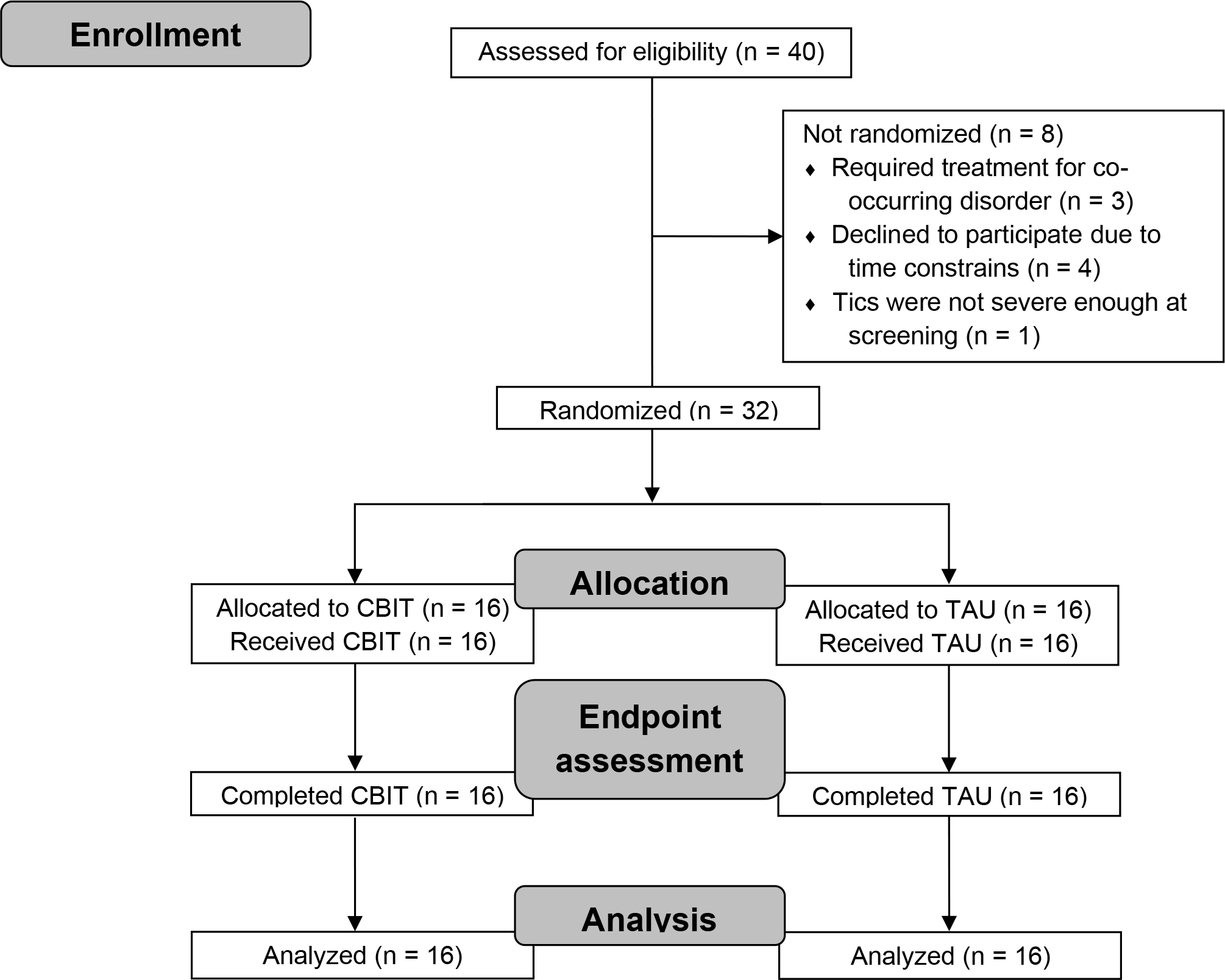


**Figure S1: Flow chart of participants recruited in the trial.** Initially published in Morand-Beaulieu et al. (2022). CBIT: Comprehensive Behavioral Intervention for Tics, TAU: treatment-as-usual.

| **Table S1: Connections involved in the tic suppression subnetwork** |  |
| --- | --- |
| Connection | |
| L postcentral gyrus – L precuneus | |
| R cuneus – L postcentral | |
| L banks of the superior temporal sulcu­­­­­s – L lingual gyrus | |
| L caudal middle frontal gyrus – L precentral gyrus | |
| L parahippocampal gyrus – L precentral gyrus | |
| L banks of the superior temporal sulcus – L inferior temporal gyrus | |
| L caudal middle frontal gyrus – R lateral occipital cortex | |
| R caudal anterior cingulate gyrus – L precuneus | |
| L inferior parietal cortex – R superior frontal gyrus | |
| L isthmus of the cingulate cortex – L posterior cingulate cortex | |
| L caudal middle frontal gyrus – R superior parietal cortex | |
| L isthmus of the cingulate cortex – R superior frontal gyrus | |
| L banks of the superior temporal sulcus – R lingual gyrus | |
| R inferior parietal cortex – R superior frontal gyrus | |
| L lateral occipital cortex – R superior frontal gyrus | |
| L precuneus – R superior frontal gyrus | |
| L parahippocampal gyrus – L transverse temporal cortex | |
| R caudal anterior cingulate cortex – L superior parietal cortex | |
| L caudal anterior cingulate cortex – L superior parietal cortex | |
| L pars triangularis – R superior parietal cortex | |
| L pericalcarine cortex – R superior frontal gyrus | |
| L postcentral gyrus – L superior parietal cortex | |
| R caudal middle frontal gyrus – L precuneus | |
| R middle temporal gyrus – R superior frontal gyrus | |
| R superior frontal gyrus – R superior parietal cortex | |
| L precentral gyrus – L precuneus | |
| R pericalcarine cortex – R superior frontal gyrus | |
| L inferior parietal cortex – L superior frontal gyrus | |
| L banks of the superior temporal sulcus – R superior frontal gyrus | |

References

Debnath, R., Buzzell, G. A., Morales, S., Bowers, M. E., Leach, S. C., & Fox, N. A. (2020). The Maryland analysis of developmental EEG (MADE) pipeline. *Psychophysiology, 57*(6), e13580. <https://doi.org/10.1111/psyp.13580>

Leach, S. C., Morales, S., Bowers, M. E., Buzzell, G. A., Debnath, R., Beall, D., & Fox, N. A. (2020). Adjusting ADJUST: Optimizing the ADJUST algorithm for pediatric data using geodesic nets. *Psychophysiology, 57*(8), e13566. <https://doi.org/10.1111/psyp.13566>

Mognon, A., Jovicich, J., Bruzzone, L., & Buiatti, M. (2011). ADJUST: An automatic EEG artifact detector based on the joint use of spatial and temporal features. *Psychophysiology, 48*(2), 229-240. <https://doi.org/10.1111/j.1469-8986.2010.01061.x>

Morand-Beaulieu, S., Crowley, M. J., Grantz, H., Leckman, J. F., Scahill, L., & Sukhodolsky, D. G. (2022). Evaluation of EEG biomarkers of comprehensive behavioral intervention for tics in children with Tourette syndrome. *Clinical Neurophysiology*. <https://doi.org/10.1016/j.clinph.2022.07.500>

Nolan, H., Whelan, R., & Reilly, R. B. (2010). FASTER: Fully Automated Statistical Thresholding for EEG artifact Rejection. *Journal of Neuroscience Methods, 192*(1), 152-162. <https://doi.org/10.1016/j.jneumeth.2010.07.015>
